# Staged Endovascular and Open Surgical Repair of Multiple High-Risk Thoracoabdominal Aortic Aneurysms Following Initial Presentation With a Ruptured Juxtarenal Aneurysm: A Case Report

**DOI:** 10.7759/cureus.87195

**Published:** 2025-07-02

**Authors:** Kenji Sakai, Yushi Okumura, Tetsuya Yoshida

**Affiliations:** 1 Department of Cardiovascular Surgery, Hokushin General Hospital, Nakano City, JPN

**Keywords:** aortic aneurysm, evar, open arch replacement, spinal cord ischemia prevention, staged repair, tevar

## Abstract

Simultaneous repair of extensive aortic aneurysms carries a high risk of spinal cord ischemia. Staged repair is one strategy to reduce this risk; however, aneurysm rupture during the interval is a concern. A 74-year-old man presented with a ruptured juxtarenal abdominal aortic aneurysm (AAA). A CT scan revealed a large (87 mm) ruptured AAA, a 95-mm descending thoracic aortic aneurysm (TAA), and an 80-mm saccular aneurysm in the aortic arch. Emergency EVAR (endovascular aneurysm repair) and right renal artery embolization were performed. Two weeks later, TEVAR (thoracic endovascular aortic repair) was completed for the descending TAA. At six weeks post-initial intervention, open arch replacement with open stent graft technique was performed. The patient had no neurologic or renal complications. Staged repair, prioritizing rupture risk, can allow the complete and safe treatment of extensive aortic disease without major complications.

## Introduction

Extensive thoracoabdominal aortic aneurysms involving multiple segments present a significant therapeutic challenge due to the risk of spinal cord ischemia (SCI), which remains one of the most feared complications following complex aortic repair, with an incidence reported as high as 9.6-21.8% in some series [1,2]. Staged repair strategies have been increasingly employed to mitigate this risk by encouraging collateral network development and allowing physiological adaptation [3]. However, most reports describe elective cases with stable patients and do not fully address urgent or ruptured scenarios involving extensive aortic disease. Here, we present a unique case of a hemodynamically unstable patient with a ruptured juxtarenal abdominal aortic aneurysm (AAA), in whom large aneurysms of the thoracic and aortic arch were also present. This case was successfully managed using a planned three-stage hybrid repair strategy, combining open and endovascular techniques across multiple aortic segments.

## Case presentation

This case report was approved by the Ethics Committee of Hokushin General Hospital (approval no. 2025007). Written informed consent was obtained from the patient.

**Figure 1:**
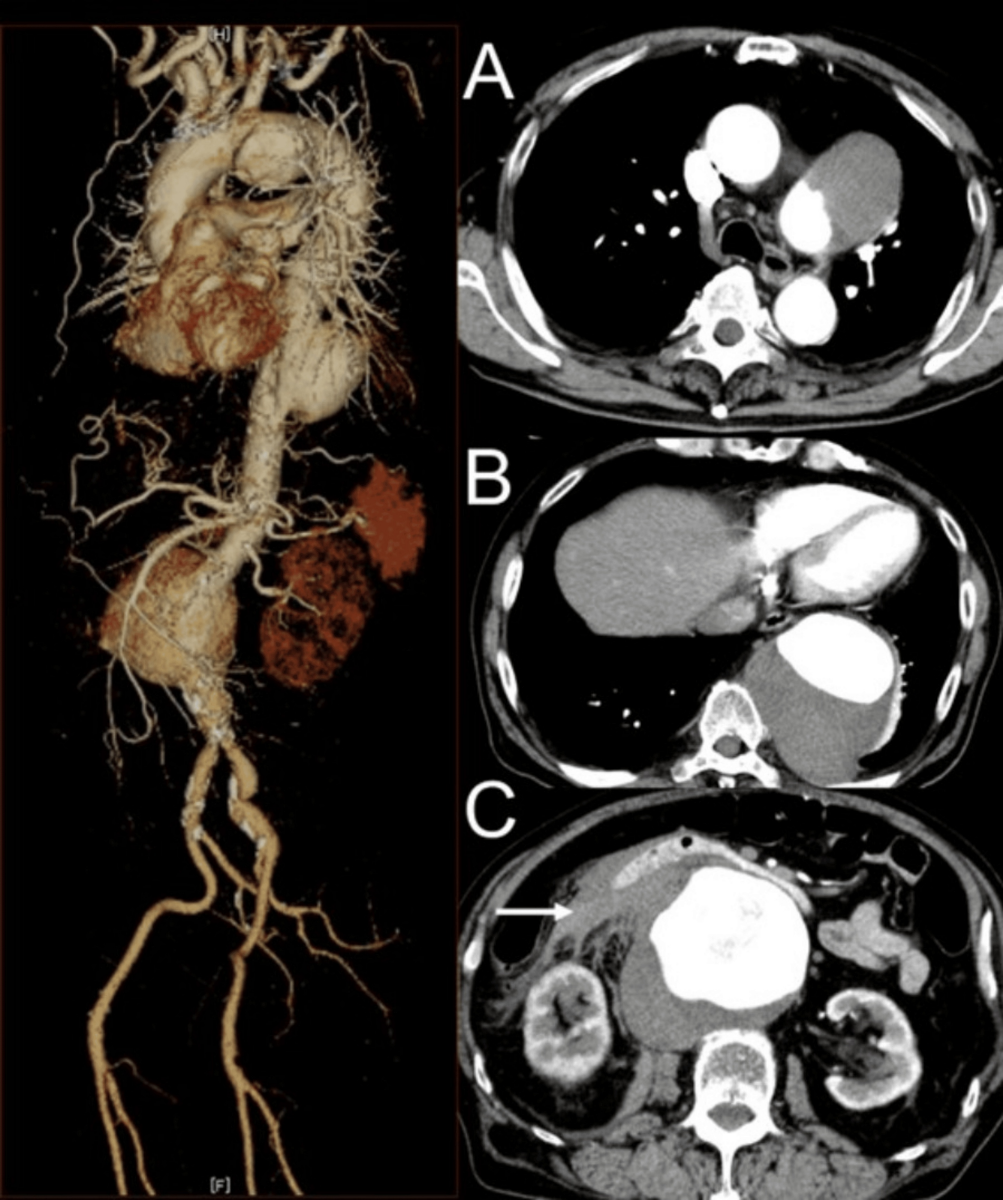
Contrast-enhanced CT findings Preoperative contrast-enhanced CT scans showing (A) an 80-mm saccular aneurysm of the aortic arch; (B) a 95-mm aneurysm of the descending thoracic aorta; and (C) an 87-mm ruptured juxtarenal abdominal aortic aneurysm with retroperitoneal hematoma (arrow).

A 74-year-old man with a history of hypertension, type 2 diabetes mellitus, and dyslipidemia presented to the emergency department with sudden-onset right upper quadrant and back pain. On arrival at the emergency department of Hokushin General Hospital (Nakano City, Nagano, Japan), he was hypotensive (blood pressure: 79/40 mmHg, heart rate: 55 bpm), with a palpable pulsatile mass in the abdomen. A contrast-enhanced computed tomography (CT) demonstrated an 87-mm ruptured juxtarenal AAA with retroperitoneal hematoma and active contrast extravasation. The main trunk of the right renal artery was compressed by the aneurysm, resulting in delayed enhancement of the right kidney. A portion of the right kidney was perfused by an accessory renal artery, which arose more proximally than the main renal artery. In addition, saccular aneurysms of the descending thoracic aorta (95 mm in diameter) and the aortic arch (80 mm in diameter) were also identified (Figure 1).

**Figure 2:**
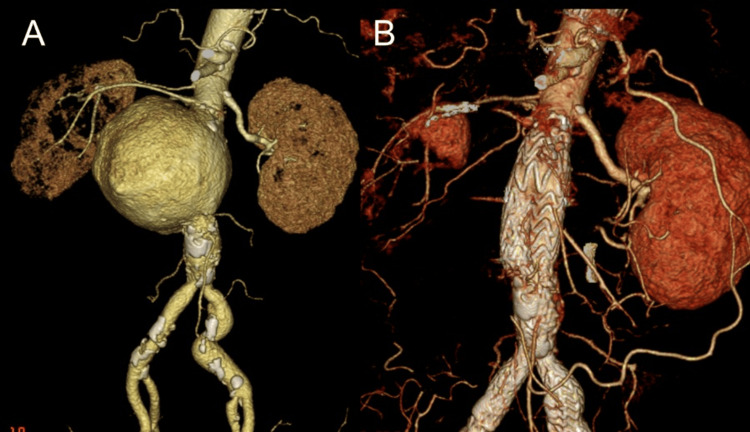
CT findings before and after emergency surgery (A) Preoperative contrast-enhanced CT showing a ruptured juxtarenal aneurysm and compression of the main trunk of the right renal artery. (B) Postoperative CT demonstrating successful aneurysm exclusion with no endoleak. The accessory renal artery is preserved, and right renal perfusion is maintained.

The first stage was performed using a GORE® EXCLUDER® device (W. L. Gore & Associates, Inc., Newark, DE, USA) for emergency endovascular aneurysm repair (EVAR), with a proximal landing zone located above the right renal artery. Given the hemodynamic instability due to rupture, securing a reliable proximal sealing zone was prioritized. The right renal artery was coil embolized, preserving the accessory renal artery (Figure 2).

Preoperative imaging revealed that the accessory renal artery provided partial perfusion to the right kidney, allowing for the embolization of the main renal artery without critically impairing renal function. The operative time was 95 minutes. The patient recovered without complications and was discharged home on postoperative day (POD) eight.

One week later, the second stage of intervention was performed using TEVAR (thoracic endovascular aortic repair) with a GORE® TAG® endoprosthesis to treat the descending thoracic aortic aneurysm (TAA). The second-stage TEVAR procedure was completed in 60 minutes without complications. The timing was selected to balance adequate recovery from the initial emergency operation and the need for prompt exclusion of the large descending thoracic aneurysm. Postoperative CT demonstrated significant shrinkage of the aneurysm sac, suggesting effective decompression and reduced risk of rupture.

**Figure 3:**
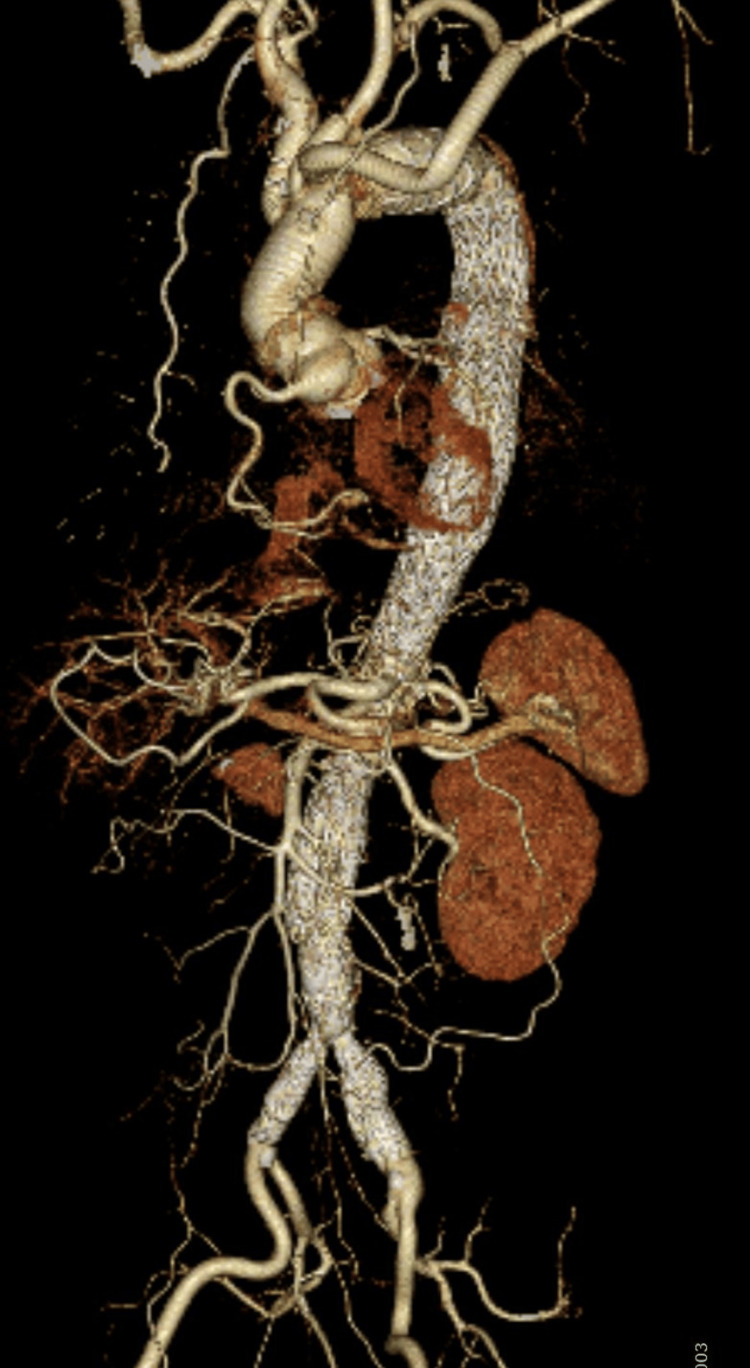
Postoperative whole-body contrast-enhanced CT No endoleak or residual aneurysm is observed. The thoracic and abdominal stent grafts are well-positioned, and the aortic morphology remains stable without any signs of migration, kinking, or sac enlargement.

Six weeks after the initial surgery, the third stage of intervention involved open total arch replacement with the open stent graft technique. The operative time was four hours and 52 minutes. To prevent paraplegia, efforts were made to correct anemia and avoid hypoxia. Prophylactic cerebrospinal fluid drainage was not performed. No neurological or renal complications were observed. The patient was discharged home on POD eight and remains under outpatient follow-up with stable renal function. Postoperative contrast-enhanced CT confirmed the absence of endoleak and successful treatment of all aneurysms (Figure 3).

## Discussion

Treatment of extensive aortic disease involving multiple aneurysmal segments presents significant challenges, especially when one of the aneurysms presents with rupture. In such cases, the clinician must balance the risk of rupture of untreated aneurysms with the danger of SCI associated with extensive or simultaneous repair. Factors influencing SCI risk include the extent of aortic coverage, interruption of critical vascular networks, intraoperative hypotension, and lack of interval remodeling. To mitigate these risks, staged repair has gained increasing support, and various treatment methods have been reported [4]. Etz et al. have shown that staged repair can significantly reduce the incidence of paraplegia by promoting gradual vascular adaptation between procedures [5].

In this case, we achieved successful aneurysm exclusion through a three-stage approach within six weeks, with no incidence of neurologic complications. Notably, the first-stage intervention was performed under emergency conditions due to rupture, which itself poses additional hemodynamic and procedural challenges. Despite this, the patient tolerated sequential interventions well, suggesting that rapid-stage repair within short intervals may be feasible and safe, provided careful hemodynamic and renal monitoring is maintained. Indeed, previous literature has indicated that even a one-week interval between staged procedures may reduce the risk of paraplegia, further supporting the validity of short-interval staged approaches [6].

The decision to coil embolize the right main renal artery while preserving the accessory renal artery was guided by preoperative anatomical assessment. In the emergent setting, securing an adequate proximal sealing zone was prioritized. Preoperative imaging demonstrated that part of the right kidney was perfused by an accessory renal artery, which allowed for the sacrifice of the main renal artery without completely compromising renal function. Postoperatively, the patient maintained stable renal function, underscoring the importance of recognizing and incorporating renal vascular anatomy, particularly the presence of accessory arteries, into procedural planning [7].

Furthermore, this case demonstrates the benefit of prioritizing aneurysms for treatment based on their rupture risk. The use of less invasive endovascular techniques in the early stages allowed rapid recovery and minimized physiologic burden, enabling a timely transition to open surgery. The open stent technique used for arch repair ensured durable fixation and preserved cerebral perfusion without neurological sequelae.

In the literature [8], there are few cases that describe successful three-segment aortic reconstruction, particularly in cases of rupture, which we were unable to find. This case supports the paradigm of individualized, anatomy-informed staged repair, suggesting that even in complex or ruptured presentations, carefully timed and anatomically guided interventions can lead to excellent outcomes.

## Conclusions

This case highlights the feasibility and effectiveness of a tailored, three-stage repair strategy for managing extensive aortic disease involving multiple aneurysmal segments, including a ruptured juxtarenal AAA. A staged approach allowed for physiologic recovery between procedures, minimized the risk of spinal cord ischemia, and facilitated appropriate prioritization of life-threatening pathology. Careful anatomical assessment, particularly of renal and collateral circulation, was essential in guiding procedural decisions. The timely use of endovascular and open techniques, based on the urgency and anatomical complexity of each case, enabled the safe and definitive exclusion of all aneurysms without neurological complications. This experience underscores the importance of individualized, anatomy-driven planning in the treatment of complex aortic pathology.
